# Copeptin Levels Remain Unchanged during the Menstrual Cycle

**DOI:** 10.1371/journal.pone.0098240

**Published:** 2014-05-27

**Authors:** Claudine A. Blum, Uzma Mirza, Mirjam Christ-Crain, Beat Mueller, Christian Schindler, Jardena J. Puder

**Affiliations:** 1 Endocrinology, Medical University Clinic, Kantonsspital Aarau, Aarau, Switzerland; 2 Endocrinology, Diabetology and Metabolism, University Hospital Basel, Basel, Switzerland; 3 Service of Endocrinology, Diabetes and Metabolism, University Hospital, Lausanne, Switzerland; 4 Swiss Tropical and Public Health Institute, Basel, Switzerland; The University of Manchester, United Kingdom

## Abstract

**Background:**

Copeptin, a surrogate marker for arginin vasopressin production, is evaluated as an osmo-dependent stress and inflammatory biomarker in different diseases. We investigated copeptin during the menstrual cycle and its relationship to sex hormones, markers of subclinical inflammation and estimates of body fluid.

**Methods:**

In 15 healthy women with regular menstrual cycles, blood was drawn on fifteen defined days of their menstrual cycle and was assayed for copeptin, progesterone, estradiol, luteinizing hormone, high-sensitive C-reactive protein, tumor necrosis factor-alpha and procalcitonin. Symptoms of fluid retention were assessed on each visit, and bio impedance analysis was measured thrice to estimate body fluid changes. Mixed linear model analysis was performed to assess the changes of copeptin across the menstrual cycle and the relationship of sex hormones, markers of subclinical inflammation and estimates of body fluid with copeptin.

**Results:**

Copeptin levels did not significantly change during the menstrual cycle (p = 0.16). Throughout the menstrual cycle, changes in estradiol (p = 0.002) and in the physical premenstrual symptom score (p = 0.01) were positively related to copeptin, but changes in other sex hormones, in markers of subclinical inflammation or in bio impedance analysis-estimated body fluid were not (all p = ns).

**Conclusion:**

Although changes in estradiol and the physical premenstrual symptom score appear to be related to copeptin changes, copeptin does not significantly change during the menstrual cycle.

## Introduction

Copeptin is increasingly evaluated as a volume- and osmo-dependent stress biomarker in different diseases. Arginin vasopressin (AVP), a hypothalamic hormone with a more subtle response to stress than cortisol [1], is difficult to measure due to its pulsatile secretory pattern, its instability and its rapid elimination from plasma [2], [3]. Copeptin derives from the prohormone of AVP, is secreted equimolarly, is stable in vitro [4] and can therefore be used as its surrogate marker [5]. Copeptin is also elevated in states of acute inflammation [1] and is being studied as a diagnostic and prognostic marker in acute and chronic illnesses [6]. It is therefore important to recognize possible confounding factors.

During the menstrual cycle (MC), there are changes in hormones involved in fluid regulation and inflammatory markers [7]–[9]. Accordingly, basal AVP plasma levels apparently undergo subtle cyclic changes, being lowest during menstruation, increasing during the follicular phase with a peak at ovulation and dropping subsequently during the luteal phase [8], [9]. There are also measurable changes in basal AVP levels in response to sex hormone treatment as seen with oral contraception [10], [11]. Dehydration and hypertonic saline infusion reduce the plasma osmolality threshold required for AVP release in the luteal phase and while taking oral contraception [12].

Body fluid can be estimated by bioimpedance analysis (BIA) [13], and symptoms related to fluid retention may be monitored, for example through a premenstrual symptoms score (PMS) [14].

Physiological hormonal and fluid changes suggest that copeptin levels may vary throughout the MC. These changes could be clinically important confounding factors if copeptin is used as a point-of-care-tool in premenopausal women with acute or chronic illness. Therefore, we investigated the changes of copeptin during the MC and its relationship to sex hormones, markers of subclinical inflammation and estimates of body fluid, such as total body fluid measured by BIA and physical PMS symptoms.

## Methods

### Ethics statement

The study adheres to the declaration of Helsinki. It was approved by the local ethics committee in Basel, Switzerland (EKBB “Ethikkommission beider Basel”), and all participants signed and received a copy of a written informed consent form before taking part in the study.

### Participants

Participants were fifteen healthy women aged 18 to 36 years with a regular MC between 26 to 33 days and confirmed ovulation by progesterone level on day 21 of the MC. Exclusion criteria were intake of oral contraceptives within the past 6 months, any medication except occasional paracetamol, smoking, diabetes, history of premenstrual syndrome, minor illness within the previous month. Physical exercise was restricted to no more than 3 hours weekly.

### Study design, definition of MC phases, and symptoms score

Women were studied during one complete MC. After an overnight fast of at least 10 hours, 30 ml blood was drawn between 0730 h and 0930 h on days 3, 5, 8–16, 18, 21, 24 and 27 of each participant's MC. Participants completed a standardized PMS questionnaire [14], [15] at every visit. Seven MC phases were defined by days from the LH peak: each cycle was first divided into a follicular phase (FP) and a luteal phase (LP) by the serum LH peak. The follicular phase was further divided into early follicular phase (EFP, days -15 to -9 from the LH peak), midfollicular phase (MFP, days -8 to -4), and late follicular phase (LFP, days -3 to -1). The luteal phase was further divided into early luteal phase (ELP, days 1–3), midluteal phase (MLP, days 4–8), and late luteal phase (LLP, days 9–14).

During EFP, MFP and MLP, patients were weighted and BIA was used to estimate total body fluid (BIA 101, Akern S. r. l., via Galeotti, 3, 50136, Firenze, Italy).

PMS was calculated based on a validated 17-item daily symptom report [14] and the factor physical symptoms score (i.e. symptoms of fluid retention: breast swelling and edema) was evaluated in the current analysis.

### Assays

The blood was immediately centrifuged, aliquoted, and stored at −70°C. Serum samples were assayed by batch analysis. For progesterone, estradiol, luteinizing hormone (LH), high-sensitive C-reactive protein (hs-CRP), tumor necrosis factor – alpha (TNF-a) and procalcitonin, standard assays were used as mentioned elsewhere [7].

Copeptin was measured by chemiluminescence sandwich immunoassay (Brahms, Henningsdorf, Germany) with a lower detection limit of the assay of 0.4 pmol/L and a functional assay sensitivity of <1 pmol/L (<20% interassay CV).

### Statistical Analysis

STATA (version 12.1 for Windows, StataCorp LP, 4905 Lakeway Drive, College Station, TX 77845 USA) was used for data analysis. Mean values for copeptin, sex hormone levels and inflammatory markers and the physical symptom score were calculated for each subject in each of the seven MC phases, also for the descriptive analyses. Where necessary, the dependent variable was log-transformed to achieve an approximately normal distribution. For instance, this was the case for copeptin. Mixed linear model analysis with compound symmetry autocorrelation structure for repeated measurements was performed to study the changes of copeptin, estradiol, progesterone, LH and the physical PMS symptoms factor across the MC phases as well as the relationship of estradiol, progesterone, LH, hs-CRP, TNF-a, procalcitonin and physical PMS factor with copeptin (as the dependent variable) across the different MC phases. This same analysis was done for the relationship of copeptin with body fluid as estimated by BIA at the EFP, MFP and MLP. In a second step, analyses were adjusted for the different MC phases to detect potential associations of copeptin with other parameters unexplained by MC-phase. We also considered models with an AR-1 autocorrelation structure which showed almost identical results (data not shown). Differences in copeptin levels between women who reached a LLP and those who did not were calculated using Wilcoxon rank-sum test. In our setting of 15 women whose copeptin levels were assessed in seven different phases across the MC, we would have had 90% power to find a statistically significant effect of the MC if the variance of this effect across the 7 time intervals were 0.2 times the residual variance (i.e., the variance of log-transformed copeptin levels within subjects unexplained by the average time effects). With a found intraclass correlation coefficient (ICC) of 0.75 (95% confidence intervals: 0.58 to 0.88), this would imply that 75% of the total variance of log-copeptin is explained by the subject (between-subject variability), 5% by the average time effects and 20% by unexplained variation within subjects. P<0.05 was considered significant.

## Results

### Changes during the MC

**Figure 1:**
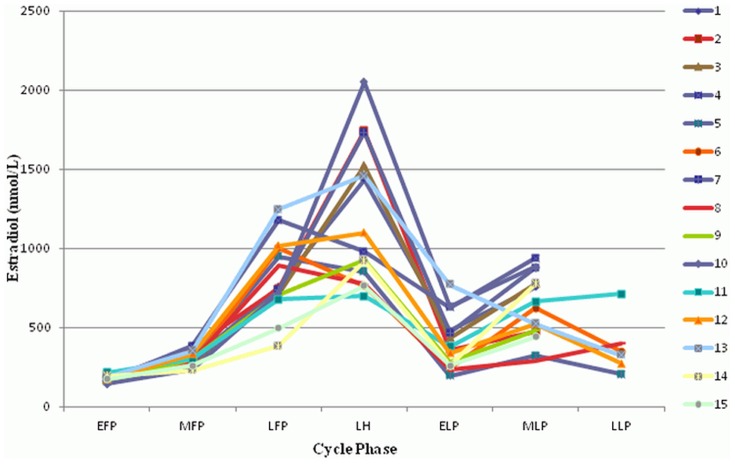
Changes of estradiol during the menstrual cycle. All 15 participants are shown (1–15). EFP: early follicular phase. MFP: midfollicular phase. LFP: late follicular phase. LH: surge of luteinizing hormone. ELP: early luteal phase. MLP: midluteal phase. LLP: late luteal phase.

**Figure 2:**
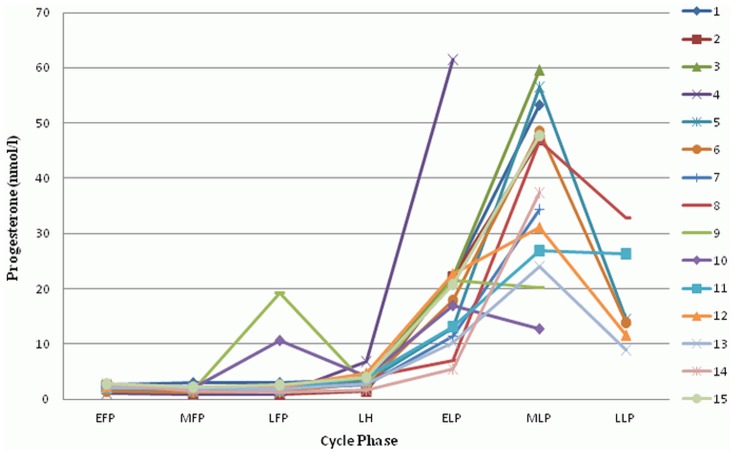
Changes of progesterone during the menstrual cycle. All 15 participants are shown (1–15). EFP: early follicular phase. MFP: midfollicular phase. LFP: late follicular phase. LH: surge of luteinizing hormone. ELP: early luteal phase. MLP: midluteal phase. LLP: late luteal phase.

**Figure 3:**
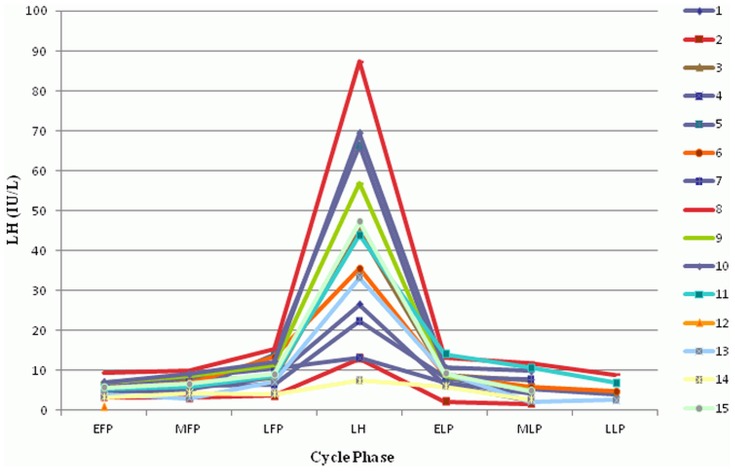
Changes of luteinizing hormone (LH) during the menstrual cycle. All 15 participants are shown (1–15). EFP: early follicular phase. MFP: midfollicular phase. LFP: late follicular phase. LH: surge of luteinizing hormone. ELP: early luteal phase. MLP: midluteal phase. LLP: late luteal phase.

**Figure 4:**
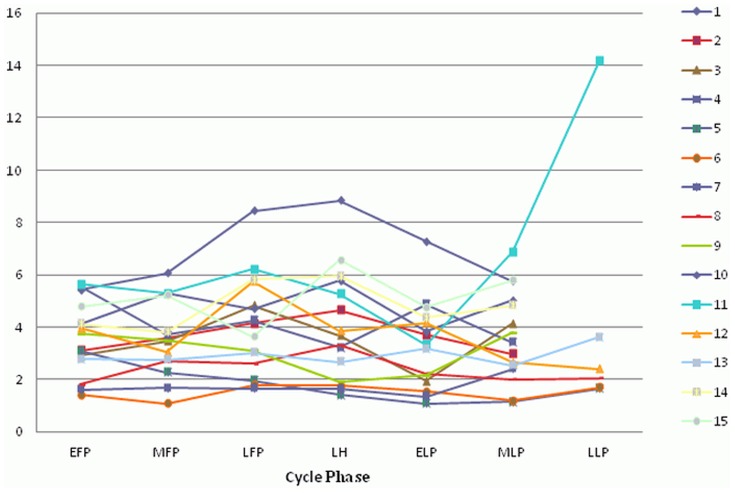
Changes of copeptin during the menstrual cycle. All 15 participants are shown (1–15). EFP: early follicular phase. MFP: midfollicular phase. LFP: late follicular phase. LH: surge of luteinizing hormone. ELP: early luteal phase. MLP: midluteal phase. LLP: late luteal phase.

Baseline characteristics are shown in table 1. Mean cycle length was 28.5+/−2.2 days. All cycles were ovulatory as confirmed by the peak serum progesterone levels. Estradiol, progesterone, and LH all showed characteristic changes during the MC (all p<0.001, Figures 1–3). Copeptin levels did not significantly change during the MC (p = 0.16, Figure 4) with a median of 3.5 pmol/l throughout the MC (interquartile range 2.3–4.8). When comparing the different MC phases using mixed linear models, highest copeptin levels were found in the LFP, LH phase and LLP, but differences to the EFP were not significant (all p>0.2) and remained within physiological limits as previously described [16] (for detailed copeptin values during each menstrual cycle phase, see table 2).

**Table 1:** Baseline characteristics.

Baseline characteristics	n = 15
Age (years)	30.9±3.3
Duration of menstrual cycle (d)	28.5±2.2
BMI (kg*m^−2^)	25.4±4.7
Hemoglobin at baseline (g %)	13.6±1.2
Peak progesterone (nmol/L)	54±14.6
Luteinizing hormone (LH) in the EFP (IU/L)	5.4 (2.2.–3.1)
Luteinizing hormone (LH) at LH-Surge (IU/L)	44.0 (24.4–58.2)
Estradiol (pmol/L)	462±361
Estradiol in the EFP (pmol/L)	166 (136–183)
Copeptin (pmol/L)	3.5 (2.3–4.8)

Normally distributed data are shown as mean ±SD, otherwise as median (interquartile range).

EFP: Early Follicular Phase.

**Table 2:** Copeptin values in the menstrual cycle.

Copeptin level (pmol/L), median (IQR)	Menstrual cycle phase
3.7 (2.8–4.8)	early follicular phase (EFP)
3.5 (2.7–5.2)	midfollicular phase (MFP)
4.1 (2.6–5.7)	late follicular phase (LFP)
3.7 (1.9–5.8)	surge of luteinizing hormone (LH)
3.3 (1.9–4.4)	early luteal phase (ELP)
3.4 (2.4–5.0)	midluteal phase (MLP)
2.2 (1.7–6.3)	late luteal phase (LLP)

Number of participants n = 15 except in LLP, which was only reached by 6 participants. The median (interquartile range) copeptin levels throughout the menstrual cycle were lower in the 6 women who had a LLP compared to the 9 women who did not (2.7 (1.8 to 3.5) pmol/L compared to 4.1 (3.3–5.2) pmol/L, p<0.05).

Physical PMS symptoms changed throughout the MC phases (p = 0.004) and were highest in the LFP. The median values overall of all MC phases were 0 (interquartile range 0–0.5), with values of the highest quartile (75^th^ percentile) being 1.25, 0.33 and 0.5 in the 3 FP, 0 in the LH phase and 0.33, 1.1 and 8.1 in the 3 LP, respectively. Total body fluid (p = 0.72) and weight (p = 0.99) did not change between the EFP, MFP and the MLP.

None of the participants developed any clinically apparent infection or other illness during the studied MC.

### Association of copeptin with sex hormones, markers of sublinical inflammation and estimates of body fluid during the MC

During the MC, the changes in estradiol were positively related to changes in copeptin levels (p = 0.002), but not changes in progesterone or LH (for detailed values, see table 3). Specifically, a change of the mean concentration of estradiol during a MC phase by 1000 pml/L was on average related to an increase in the geometric mean of copeptin by 22%.

**Table 3:** Relationship of sex hormones, inflammatory markers and estimates of body fluid with copeptin throughout the menstrual cycle.

Parameter	Beta - coefficient (95% confidence interval)	p-value
Estradiol (pmol/L)	0.0002 (0.0001–0.0003)	0.002
Progesterone (nmol/L)	−0.0014 (−0.0048–0.0020)	0.414
Luteinizing hormone (IU/L)	0.0015 (−0.0019–0.0049)	0.385
high sensitive C-reactive Protein (mg/L)	−0.02836 (−0.1243–0.0675)	0.562
Tumor necrosis factor alpha (pg/mL)	0.0485 (−0.0338–0.1309)	0.248
Procalcitonin (ng/L)	0.0002 (−0.0102–0.0105)	0.976
Total body fluid estimate by BIA (L)	0.0271 (−0.3241–0.0866)	0.372
Weight (kg)	0.0124 (−0.0029–0.0276)	0.111
Physical symptoms factor of the PMS score	0.1417 (0.0333–0.2501)	0.010

Beta-coefficient quantifies the relationship between the changes of the respective parameters with changes in copeptin concentrations (pmol/L) throughout the menstrual cycle. Copeptin concentrations were log-transformed.

BIA: bio impedance analysis.

PMS: Premenstrual symptoms score.

Changes in hs-CRP, TNF-a or procalcitonin levels were not related to changes in copeptin levels throughout the MC (see table 3).

Changes in total body fluid as estimated by BIA during the EFP, MFP and MLF or in body weight were not related to copeptin levels (Table 3). However, physical PMS symptom changes per MC phase were positively related to changes of copeptin during the MC (p = 0.01). On average, a change of the mean physical PMS symptom by 1 score was related to an increase in the geometric mean of 15%. All these results remained unchanged when the relationships between the other parameters and copeptin were additionally adjusted for the different MC phases.

## Discussion

In our cohort, we did not find significant MC-related changes of copeptin levels or any relationship to progesterone or LH, but changes of copeptin were related to estradiol during the MC, although the relationship was weak. Copeptin levels in the LFP were higher than in the EFP, but differences were not significant (p = 0.19, data not shown) and within the reported inter- and intraindividual variance in healthy women [4].

Subtle changes of AVP itself during the MC have only been found by those studies that measured at multiple time points, even with a low number of study participants ranging from 6 to 14 [8], [17], [18]. As healthy women with estrogen–progesterone-treatment were shown to have an increased risk of developing exercise-induced hyponatremia [12], estrogen-related changes in AVP may be of clinical relevance in patients taking oral contraception.

Our data on copeptin is therefore in accordance with the literature on AVP, which shows that hormones implicated in fluid regulation undergo subtle changes during the MC, appear to be influenced by estradiol, and are highest in the follicular phase [8], [17], [18], although this effect was only significant in one study [8]- Possibly, endogenous estradiol levels of the MC are not high enough or only elevated during a too short period of time to have a relevant influence on copeptin levels. In a larger cohort of healthy premenopausal women with regular MC, these subtle changes in copeptin might become statistically significant, but changes remained within physiological levels and were not related to body weight or total body fluid.

We excluded subjects with premenstrual syndrome. Nevertheless, perceived changes of body fluid as assessed by the physical symptoms of the PMS score (fluid retention) were significantly related to copeptin. However, estimates of body fluid measured by BIA did not. This may on one hand be due to the small fluid changes throughout the MC in healthy women, and on the other hand due to not assessed confounding factors [19], such as hydration status, salt intake, and a reported low validity of the BIA measurement for predicting body fluid [20].

Although copeptin levels are elevated in inflammatory states [6], we could not show a relationship between markers of subclinical inflammation and copeptin levels. Subclinical changes of inflammatory markers may be too subtle to have an influence on copeptin levels; furthermore, women with acute or chronic inflammatory states were excluded from participating.

The major limitation of this study is the small number of participants, which is in part compensated by serial hormone measurements. Possibly, in a larger sample, subtle MC-related changes might become apparent. However, such subtle changes within the inter- and intraindividual variance will probably not be of clinical importance.

Second, only six women had measurements in the LLP due to the variability of MC length.

Third, the data are only valid for morning measurements, as potential circadian changes of copeptin are not taken into account.

## Conclusions

We did not find any significant changes of copeptin levels during the MC in healthy regularly menstruating women, although we found a weak relationship between estradiol and copeptin. During the MC, subtle changes of body fluid assessed by physical symptoms of fluid retention were related to copeptin, but markers of subclinical inflammation were not.

## Supporting Information

Table S1 **Estradiol.** Estradiol values used for creating figure 1 are shown for each participant (1–15) and menstrual cycle phase, calculated as mean values from all measurements within the corresponding menstrual cycle phase. EFP: early follicular phase. MFP: midfollicular phase. LFP: late follicular phase. LH: surge of luteinizing hormone. ELP: early luteal phase. MLP: midluteal phase. LLP: late luteal phase.
(XLSX)

Table S2 **Progesterone.** Progesterone values used for creating figure 2 are shown for each participant (1–15) and menstrual cycle phase, calculated as mean values from all measurements within the corresponding menstrual cycle phase. EFP: early follicular phase. MFP: midfollicular phase. LFP: late follicular phase. LH: surge of luteinizing hormone. ELP: early luteal phase. MLP: midluteal phase. LLP: late luteal phase.
(XLSX)

Table S3 **Luteinizing Hormone.** Luteinizing hormone (LH) values used for creating figure 3 are shown for each participant (1–15) and menstrual cycle phase, calculated as mean values from all measurements within the corresponding menstrual cycle phase. EFP: early follicular phase. MFP: midfollicular phase. LFP: late follicular phase. LH: surge of luteinizing hormone. ELP: early luteal phase. MLP: midluteal phase. LLP: late luteal phase.
(XLSX)

Table S4 **Copeptin.** Copeptin values used for creating figure 4 are shown for each participant (1–15) and menstrual cycle phase, calculated as mean values from all measurements within the corresponding menstrual cycle phase. EFP: early follicular phase. MFP: midfollicular phase. LFP: late follicular phase. LH: surge of luteinizing hormone. ELP: early luteal phase. MLP: midluteal phase. LLP: late luteal phase.
(XLSX)
